# Protocol for capturing 3D facial meshes for rhinoseptoplasty planning

**DOI:** 10.1016/j.bjorl.2023.101289

**Published:** 2023-07-05

**Authors:** Taíse Leitemperger Bertazzo, Marcos Cordeiro D’Ornellas

**Affiliations:** a Universidade Federal de Santa Maria (UFSM), Centro de Ciências da Saúde (CCS), Programa de Pós-Graduação Mestrado Profissional em Ciências da Saúde, Santa Maria, RS, Brazil; b Universidade Federal de Santa Maria (UFSM), Departamento de Computação Aplicada em Saúde, Santa Maria, RS, Brazil

**Keywords:** Rhinoplasty, Photogrammetry, Three-dimensional image

## Abstract

**Objectives:**

To present and execute a protocol for the capture of 3D facial images using photogrammetry through the open access software Blender and its add-on OrtogOnBlender (OOB) and to evaluate the compatibility of the 3D meshes generated with Computed tomography (CT) of the sinuses.

**Methods:**

Individuals >18 years old, candidates for Rhinoseptoplasty in a tertiary hospital, were submitted to a photographic session to perform the standardized protocol. In the session, divided into 3 phases, sequential photos were taken for processing the photogrammetry in the OOB and producing 3D meshes of the face. The photogrammetry reconstructions were compared with the reference mesh of the soft tissue surface of the Sinus CT scan to assess compatibility between them.

**Results:**

21 patients were included, 67% female. 3 photogrammetry meshes and 1 CT reference mesh were generated, which demonstrated matching compatibility, as most of the mean distances between cloud points were <1.48 mm. Phase 3 of the session with the highest number of photos (54.36 ± 15.05) generated the most satisfactory mesh with the best resolution.

**Conclusions:**

The proposed protocol is reproducible and feasible in clinical practice, generated satisfactory 3D meshes of the face, being a potential tool for surgical planning and comparison of results. For the implementation of photogrammetry for use in 3D anthropometry, it is necessary to validate this method.

**Level of evidence:**

3.

OCEBM Levels of Evidence Working Group.^1^ "The Oxford 2011 Levels of Evidence". Oxford Centre for Evidence-Based Medicine. http://www.cebm.net/index.aspx?o=5653

## Highlights

• 3D Anthropometry is an effective and safe method for measuring facial distances.
• Photogrammetry is performed quickly and non-invasively.
• Photogrammetry allows measurements, surgical simulation, and prediction of results.
• Photogrammetry by OrtogOnBlender (OOB) is affordable and cost-effective.
• OOB produces satisfactory meshes for Rhinoplasty planning.

## Introduction

Photographic analysis is a key component of the examination of individuals undergoing facial aesthetic procedures, as it serves as a visual aid in the process of communication and alignment of expectations between patient and surgeon.^1^ Knowledge of photographic patterns and facial aesthetic analysis allows documentation, evaluation of results and promotion of scientific content, as well as better systematization of knowledge for doctors in training.^2^

In addition to the photographic record, anthropometric and facial asymmetry assessments are crucial, especially in Rhinoplasty, as the nose is the central organ of the face and has great implications for defining proportionality.^3^ For this, different methods were developed, such as direct and indirect anthropometry, cephalometry and Three-Dimensional (3D) anthropometry. In the last 20 years, there has been an increase in the use of 3D anthropometry, as it has proven to be an effective and safe method for measuring facial measurements. In this context, several non-invasive techniques have been designed to capture facial topographic surface data, including stereophotogrammetry, photogrammetry and laser^4^ surface scanners.

Stereophotogrammetry consists of using equipment to capture simultaneous images from different positions and angles to obtain coordinates and 3D reconstruction of the face using triangulation by convergent arrangement of the equipment's cameras. As validated in previous studies,^5^, ^6^, ^7^, ^8^, ^9^ it is a method with good accuracy and reproducibility to measure distances and volumes on the face, in addition to being able to obtain texture and color of the captured object. However, the high cost of the equipment needed for this 3D photography technique, which can vary from US$ 10,000.00 to US$ 40,000.00, is a limiting factor for its popularization.^10^

In photogrammetry, data capture is performed from a sequence of photographs obtained from different points of view, making it more accessible for use in clinical and surgical routine. During processing, a software performs the three-dimensional alignment of the captured images based on the detection of overlaps and equivalent points, thus determining the position and orientation of the camera relative to the photographed face. From this orientation, the triangulation of several detected points is carried out, thus restoring the 3D facial geometry, in the form of a point cloud or three-dimensional mesh.^11^, ^12^, ^13^ This method captures images of the face quickly and non-invasively, allows measurements of anthropometric parameters, surgical simulation and prediction of results.^14^

The objectives of this work are to present and execute a facial 3D image capture protocol with easy-to-use tools and open access; to compare the 3D meshes generated by photogrammetry with the 3D meshes of the Sinus of the Face Computed Tomography in order to analyze the compatibility between the 3D facial reconstructions and to demonstrate the potential usefulness of these methods in the planning of rhinoseptoplasty.

## Methods

### Design and participants

The work was carried out at the Otorhinolaryngology outpatient clinic of a tertiary teaching hospital, from November 2020 to December 2021. Candidates for aesthetic and/or functional rhinosepoplasty were included, of both sexes, over 18 years old and who had already undergone Computed Tomography (CT) of the Sinuses, as routinely requested in the Service. Exclusion criteria were: 1) Patients with craniofacial congenital anomalies; 2) History of head and neck trauma and/or tumors; 3) Patients with central and/or peripheral neurological disorders. In addition, specialists in facial aesthetic surgery and one of the developers of the photogrammetry tool used analyzed the results obtained.

The project was approved by the Ethics and Research Committee under number 055889 and registered at Plataforma Brasil under number CAAE 47922621.5.0000.5346. The Informed Consent Form (TCLE) was applied and explained to the volunteers, before their inclusion. Due to the global scenario, all procedures performed followed the safety protocols in force due to the SARSCoV2 pandemic.

### Image capture protocol

A protocol for setting up a photographic studio was developed; definition of lighting parameters, distance from the patient and systematization of image capture for 3D photogrammetry using the open source software Blender and its OrtogOnBlender (OOB) tool.

Also, we performed a comparative analysis of the 3D meshes generated by photogrammetry and surface reconstruction of soft tissues from Sinus CT scan to verify their compatibility in the CloudCompare software.

### Assembly of the photographic studio and standardization

The studio was set up in an appropriate room with control over quality and reproducibility factors. The lighting contained artificial and constant light with a single fluorescent lamp centered on the ceiling of the room, with a neutral temperature; and an LED illuminator, ring light model, 26 centimeters (cm) in diameter. There was minimal interference from external ambient light. We used the photographic background in sky blue, as it can be used for any ethnic type and for color or black and white photographs, according to the definitions of the Clinical Photography Committee of the Plastic Surgery Educational Foundation.^15^

### Photographic session

Sequential photos were taken, with the patient in the neutral frontal position, with eyes closed to minimize movement during capture. The distance between the examiner and the patient was 50 cm, as defined in the official protocol of the OOB documentation by. ^38^ The distortion effects that occur in two-dimensional photographs when inadequate focal lengths are used, such as the “fisheye” effect, do not affect the 3D reconstruction, since the software uses as a basis the triangulation of common points in the photographs to produce the mesh. Furthermore, if the capture is done in an open field, a significant part of what is behind the photographed individual will be digitized, which increases image noise and reconstruction quality.

For the acquisition of precision photogrammetry, an Apple smartphone, model iPhone 11, was used, as it has mechanisms for image acquisition by infrared, a TrueDepth camera system that guarantees intuitive and secure authentication, in addition to mapping the geometry of the face precisely. The iPhone 11 smartphone was chosen for the characteristics of the camera technology, which is dual, having an ultra-angle lens and a 12-Megapixel (MP) wide-angle lens. The ultra-wide aperture is f/2.4 and has a 120° field of view, while the wide angle has an f/1.8 aperture and provides optical image stabilization. In addition, it features True Tone Flash with brighter and slow sync, automatic image stabilization and captured image formats are HEIF and JPEG.^16^ It is worth remembering that the collections began in 2020, so there are currently more advanced and higher resolution smartphone models, which may have different results.

**Figure 1:**
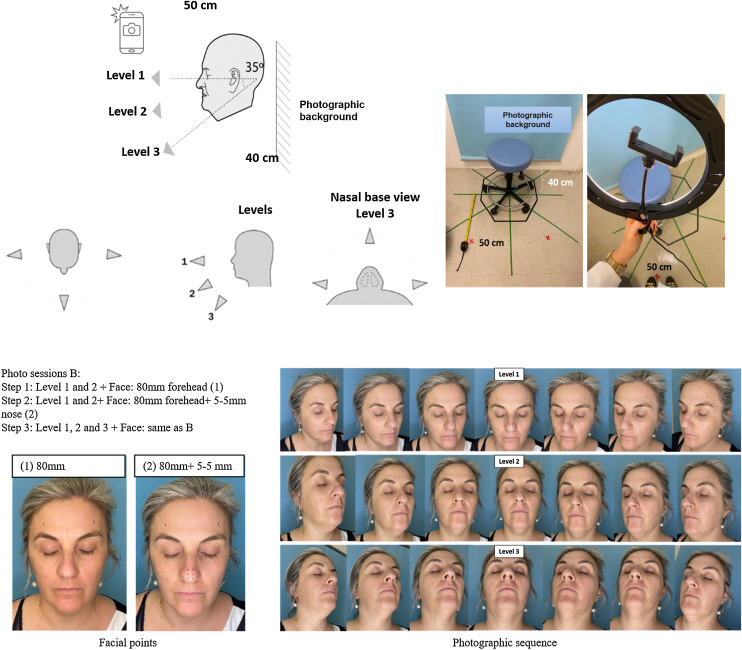
Patient and examiner positioning in the session. facial markings and capture levels of the photographic sequence phases. Note: The photographic sequence only illustrates the capture levels, not the number of captures per phase.

We divided the session into 3 phases, which are differentiated by the number and levels of photos captured, in addition to facial markings (Fig. 1), to verify if there are changes in the result of the reconstructions and to demonstrate a simpler alternative of capture in relation to the official protocol of the software. In phase 1, we captured photographs at 2 different levels with 80-millimeter (mm) forehead marking; in phase 2, there were 2 levels with an 80 mm marking on the forehead and nasal points 5 mm apart; in phase 3, there were 3 capture levels with the same facial points as in phase 2.

### Processing and comparisons of 3D meshes

The image processing was done in OOB, by the OpenMVG + OpenMVS gold standard photogrammetry algorithm defined by the developers of the OOB tool. The reconstruction of phase 1 was called “photo 1”; from phase 2 of “photo 2” and from phase 3, “photo 3”. The sinuses CT multislice images were reconstructed by the OOB SLICER algorithm, being defined as the reference mesh. All meshes produced were stored in STL (Standard Triangle Language) and OBJ (Object File Wavefront 3D) format.

The comparative analysis was performed with an ICP (Iterative Closest Point) algorithm using the Cloud Compare (CC) software version 2.9.1 (GPL, 2020), which automatically creates a cloud of points in the reconstructions, in which the number of points (Value) is defined by the resolution of the scans that generated the 3D image. The pairing of the reference mesh (TC) with the photogrammetry meshes made by the ICP is based on the “nearest neighbor distance” and the comparisons from Cloud to Mesh (C2M) evaluate the compatibility between the different captures.^17^ The C2M distance is calculated as the absolute Hausdorff distance,^18^ and is projected onto the target mesh using a “heat map”, warmer colors represent increased distances and cooler colors low distance values. These results were analyzed by two experts and one of the OOB developers.

### Statistic

For small samples (n < 30) it is recommended to use non-parametric tests, as they do not require assumptions regarding data distribution. In this case, to test whether there is a difference between several related groups, we use Friedman’s Anova, which is used when there are more than two conditions and the same participants contributed to all of them. Descriptive statistical analyzes were performed using Microsoft Excel version Office 2013 and non-parametric statistical tests using Jamovi software version 2.3 (Retrieved from https://www.jamovi.org).

## Results

**Figure 2:**
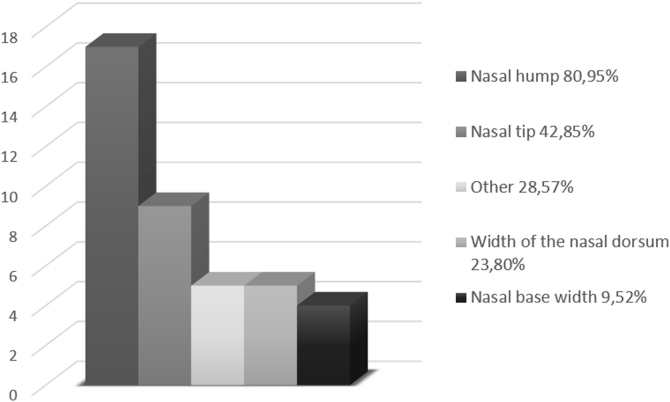
Frequency of nasal aesthetic complaints.

Twenty-one patients who were candidates for aesthetic and/or functional rhinoseptoplasty at the study’s Otorhinolaryngology Service were included, most of them female (67% — 14); with a mean age of 35.6 ± 13.8 years and a maximum of 67 years. Most patients had the presence of a hump on the nasal dorsum as their main complaint of aesthetic dissatisfaction (80.95%) (Fig. 2).

During the session, sequential photos were captured for photogrammetry processing in the OOB, generating 3 3D meshes for each patient (Table 1). Phase 3 (photo 3) had the highest average number of photos (54.36 photos) and consequently the longest time for processing (25.17 min). Phase 1 (photo 1) had an average of 36 photos with an average reconstruction time of 16.53 min and phase 2 (photo 2), 37 photos and 16.94 min. The CT scan of the sinuses was also produced at the OOB and presented a processing time interval of 1.39 min to 2.94 h, with a median of 4.96 min, this large variation is justified by the different sources of the images of CT and the longer software calibration time in the first reconstructions performed.

**Table 1:** Number of photographs and processing time in the 3 phases of the session carried out to produce 3D meshes through photogrammetry in OOB.

Session	Number of photographs (mean/standard deviation)	Processing time in minutes (mean/standard deviation)
Phase 1(photo 1)	36.47 ± 5.79	16.53 ± 0.96
Phase 2 (photo 2)	37.42 ± 11.88	16.94 ± 6.65
Phase 3 (photo 3)	54.36 ± 15.05	25.17 ± 7.50

**Figure 3:**
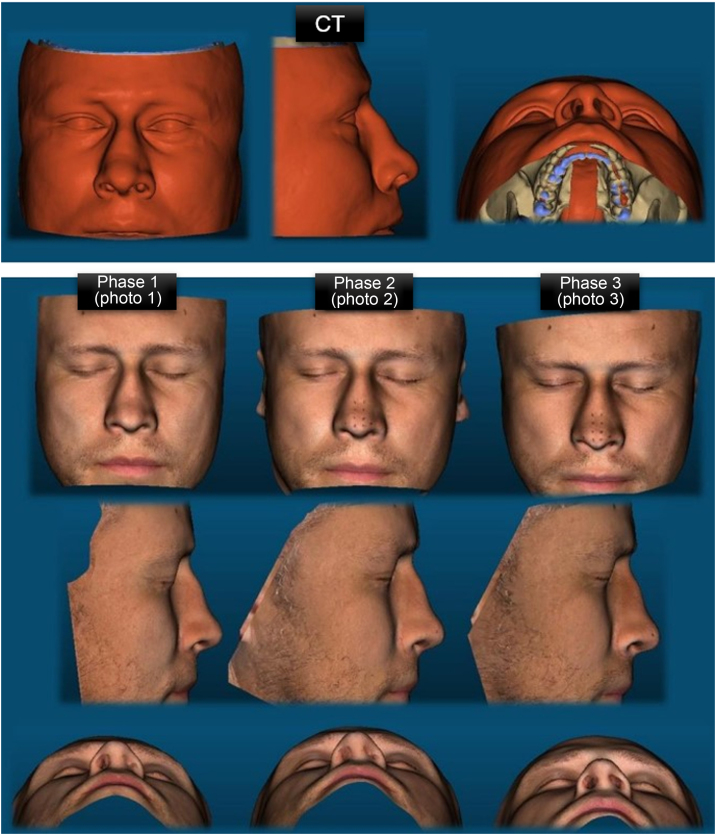
Representation of the 3D meshes produced in a male patient, reconstructed from the CT of the facial sinuses and by photogrammetry in the different capture phases.

**Figure 4:**
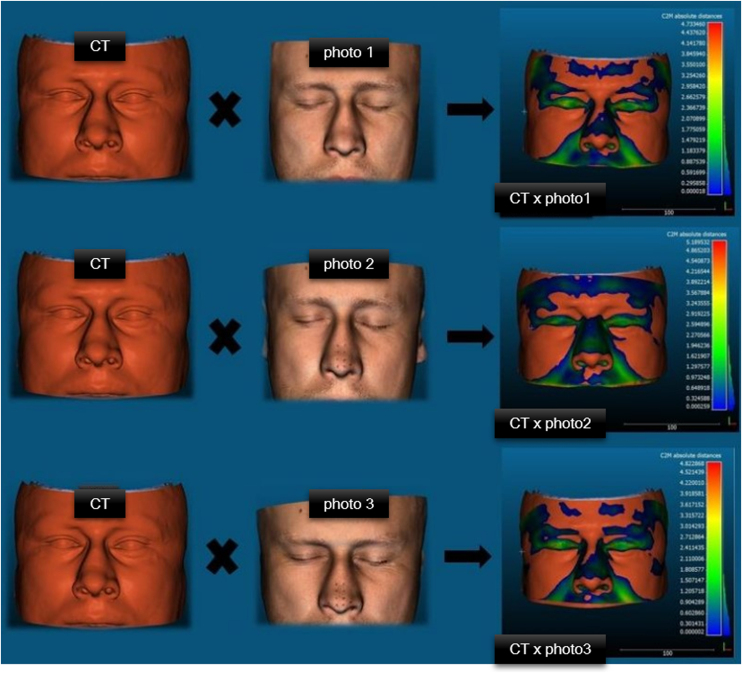
Representation of heat maps resulting from Cloud-to-Mesh (C2M) comparisons of pairings with the absolute mean distance in a male patient.

**Figure 5:**
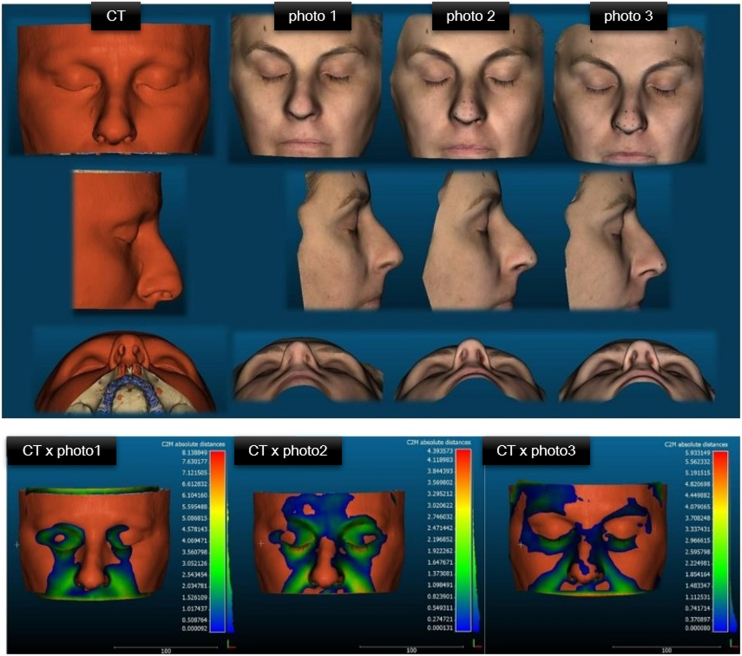
Representation of CT reconstructions of the facial sinuses and phases 1, 2 and 3 of photogrammetry in a female patient. Below, representation of heatmaps resulting from Cloudto-Mesh (C2M) comparisons of pairings with absolute mean distance.

Comparison of pairs of 3D meshes (CT versus photogrammetry) was based on the ICP algorithm applied by the CloudCompare software and resulted in 3 pairings for each patient (Fig. 3). CC automatically produces descriptive statistical data to measure compatibility between paired meshes and produces representations of pairings with heat maps (Figure 4, Figure 5). Warmer colors represent increased distances between samples, i.e., greater variation, and cooler colors represent low distance values, i.e., the target sample is a close match to the reference sample.

**Figure 6:**
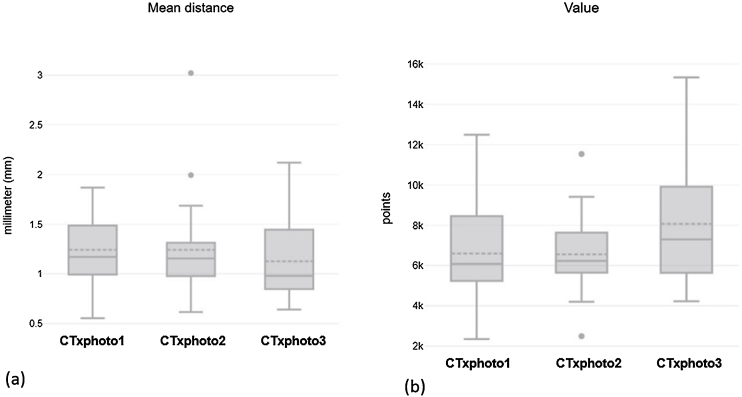
Graphs representing the pairings between CT and photogrammetry of (a) number of vertices or points (Value) considered in each pairing and (b) average distance between each vertex between the meshes (Mean distance). Caption: Friedman test was performed on both variables (a) and (B), there was no statistically significant difference between the pairings.

The reconstruction of phase 3 (photo 3) showed a range of values from 4232 to 15,331 vertices in the mesh pairing (MD = 8070; DP = 3074), representing the photogrammetry group with the highest density of points and the best resolution (Table 2). Regarding the average distance between the vertices (Mean_dist) of the clouds of paired points (Table 3), the Friedman Test was performed, which did not show a significant difference between the groups (x²(2) = 1.24; *p* > 0.05). In the sample, this mean distance ranged from 0.55 to 1.87 mm (Median 1.17) at CTxphoto1, 0.61–3.02 mm (Median 1.16) at CTxphoto2 and 0.64–2.12 mm (Median 0.98) at TCxfoto3. We observed that 75% (Q3) of the pairing values in photo 1 were below 1.48 mm, in photo 2 <1.31 mm, in photo 3 <1.42 mm (Fig. 6).

**Table 2:** Number of vertices between pairings of 3D photogrammetry and sinus CT meshes.

Number of vertices	CTxphoto1	CTxphoto2	CTxphoto3
Mean	6597,38	6555,62	8070,19
Median	6084	6235	7303
Standard deviation	2258,85	1997,57	3073,85
Minimum	2355	2497	4232
Maximum	12486	11534	15331

**Table 3:** Mean distance (in millimeters) between the pairing vertices of the 3D photogrammetry and sinus CT meshes.

Mean distance	CTxphoto1	CTxphoto2	CTxphoto3
Mean	1.24	1.24	1.13
Median	1.17	1.16	0.980
Standard deviation	0.356	0.508	0.398
Minimum	0.554	0.615	0.641
Maximum	1.87	3.02	2.12
Q1	1.01	0.995	0.863
Q2	1.17	1.16	0.980
Q3	1.48	1.31	1.42

## Discussion

Alignment between patient and surgeon expectations is essential for a satisfactory outcome. The use of 3D technology facilitates this communication by allowing a more realistic view of the face with analysis of proportions and also by enabling the simulation of results according to the patient's preferences. Rudy et al.^19^ presented some limitations for the widespread adoption of 3D images in clinical practice, among them are the lack of familiarity of surgeons with the capture and processing of 3D meshes and the high cost of validated equipment. Sobral et al.^20^ published a study presenting the use of OOB as a simple and low-cost method for capturing images and 3D simulation for rhinoseptoplasty. These authors concluded that the method is an excellent option that offers proven usefulness, accessibility, and ease of use.

In this work, we used OOB technology to create 3D facial meshes and verified its compatibility with sinus CT reconstruction. Rocha et al.^21^ demonstrated that it is possible to establish craniofacial measurements with adequate precision and defined a standard for quantitative analysis of 3D CT images, using high resolution images with the technique of volume rendering by computer graphics. We used CT reconstruction of the paranasal sinuses as a reference due to the availability of the exams, as they are routine in the study service and, in addition, due to the confirmation in previous studies of the reliability and accuracy of CT for measuring facial soft tissue thickness.^22^, ^23^, ^24^, ^25^

Assessing the accuracy of the 3D reconstruction method is the critical element and there are useful tools such as geometric surface comparison. Two 3D facial models can be aligned so that differences in facial contour and reconstruction can be numerically computed, in addition to providing a spatial map of differences in each reconstructed region.^26^, ^27^, ^28^, ^29^ Early studies in the area of forensic 3D three-dimensional anthropometry^30^, ^31^ described a type of precision threshold for the clinical use of these instruments, defining that discrepancies in facial structures smaller than 1.5 mm could be accepted, since smaller values are generally not discriminated by the naked eye. Other studies that evaluated the 3D measurement error with different facial scanning systems demonstrated the performance of facial scanners with a mean deviation of less than 2 mm.^32^, ^33^, ^34^, ^35^ Some authors suggest that deviations of less than 2 mm are considered acceptable in studies of linear distance of anthropometric points.^36^

Our quantitative approach calculated the absolute distances between two scans and provided a measure of fidelity between the captured meshes and the reference using the CloudCompare software. This software measures the Euclidean distance between the closest points, which are not necessarily the facial anthropometric points, therefore it evaluates the distance between the meshes in a holistic way. Our results in Table 3 show that most of the mean distance values between the paired grid points were < 1.5 mm, thus confirming the compatibility of photogrammetry with CT reconstruction. It is worth noting that the soft tissue thickness of an individual will never be completely accurate, as the tissue thickness markers used are averages, as concluded by Short et al.^37^ in their studies.

The results were analyzed by two otorhinolaryngologists who are specialists in facial aesthetic surgery with up to 15 years of experience and who routinely use 3D technology. There was consensus that the proposed protocol efficiently meets the production of a 3D mesh with photorealistic texture suitable for the virtual planning of surgeries and implementation in medical teaching, however, some caveats were pointed out. The specialists considered the phase 3 photogrammetry (photo 3) to be more satisfactory, the nasal base and the nostrils were the regions with the lowest visual quality and considered all reconstructions by photogrammetry to be reasonable. They point out that this software is already used by many surgeons, but it still does not have scientific validation, therefore depending on further studies with already validated instruments. In addition, one of the OOB developers found inconsistencies and low visual quality in some reconstructions, which he related to protocol modifications in the addon's official documentation, such as the framing and focal length of the photos, the lighting, and the marking of facial points.

In recent studies, the OOB developers identified flaws in the standard photogrammetry defined in the official documentation, mainly in the nasal region, since the nose has a peculiar shape, with recessed areas with shading, straight areas with light reflection and areas with no of structures, such as the nostril openings.^38^ Thus, it was proposed to capture a third sequence of photographs with emphasis on the nasal base, and also to increase the number of images collected at each level.^39^ The greater clarity of the reconstructions with the modifications of the official protocol was evidenced by the specialists in this project, who considered the 3D facial reconstruction of phase 3 of better visual quality in relation to the other photogrammetry sessions and, quantitatively, 52.63% of the average distances between phase 3 and reference meshes were < 1 mm.

In addition to the production of 3D facial meshes, OOB has tools for virtual simulation and production of surgical guides, as demonstrated by Sobral et al.^20^ The virtual facial molding is performed with the alignment and resizing of the 3D mesh, requiring the measurement of a measurement directly on the patient with a caliper, which is why, in our study, the fixed measurement of 80 mm on the forehead. Afterwards, anthropometric anatomical points are marked, and, with Blender's sculpting tools, it is possible to mold the desired shape of the nose. Surgical guides can be automatically created within the RhinOnBlender add-on based on the midline points of the face and can be exported in STL format for 3D printing. The 3D guide can be produced, for example, through melt deposition modeling with Polylactic Acid (PLA) filaments, which is an autoclavable material.

In this article, a 3D image capture protocol was presented through the use of accessible and economically viable tools for the dissemination of three-dimensional technology in the context of the preoperative evaluation of facial aesthetic surgeries. The search for more accessible alternatives drives the popularization of 3D images and their diffusion in the medical field, hence the importance of studying new tools to define their merits and weaknesses.

## Conclusion

We conclude that photogrammetry through OOB produces quality 3D meshes compatible with the facial surface CT reconstruction. The described and executed protocol is feasible in clinical practice, according to the analysis of specialists. These alternative methods have shown potential utility in three-dimensional anthropometry of the face and the development of studies for scientific validation of the method is essential.

## Conflicts of interest

The authors declare no conflicts of interest.
